# Pathological Insights: Placental and Umbilical Cord Changes in Stillbirth

**DOI:** 10.7759/cureus.91471

**Published:** 2025-09-02

**Authors:** Aishwarya SR, Sujata Siwatch, Nandita Kakkar, Reetu Kundu, Neelam Aggarwal, Shiv Sajan Saini, Kirti Gupta

**Affiliations:** 1 Obstetrics and Gynecology, Postgraduate Institute of Medical Education and Research, Chandigarh, IND; 2 Histopathology, Postgraduate Institute of Medical Education and Research, Chandigarh, IND; 3 Cytology and Gynaepathology, Postgraduate Institute of Medical Education and Research, Chandigarh, IND; 4 Pediatric Medicine, Postgraduate Institute of Medical Education and Research, Chandigarh, IND

**Keywords:** autopsy, histopathology, membranes, perinatal pathology, placenta, stillbirth

## Abstract

Background: Stillbirth evaluation plays a crucial role in the management of future pregnancies, as it can help identify risk factors that may recur. Additionally, it may provide a definitive cause of death, helping bring closure to the family and guiding both emotional healing and clinical care.

Aim: To study various pathological findings of the placenta and umbilical cord in cases of stillbirths.

Participants and methods: Fifty women suffering stillbirths occurring at ≥ 24 weeks of gestation were included in the study. Demographic details, past obstetric history, and antenatal history of the present pregnancy were recorded for all women. Detailed general physical, obstetric, and systemic examinations were done. All antenatal investigations were noted. After delivery, the stillborn was weighed, and any gross congenital malformations (CMF) were documented. Histopathological evaluation was done as per the Amsterdam placental group workshop consensus statement.

Results: The majority of women were between 19 and 30 years old. A history of stillbirth in previous pregnancies was present in four women. Most of the stillbirths were antepartum. Prominent clinical causes of stillbirths included fetal malformations, fetal growth restriction, and hypertensive disorders in pregnancy. However, a notable number remained unclassified (18%). Gross and microscopic findings of the placenta, cord, and membranes are presented. The most common histopathological placental finding was fibrin deposition, seen in 38 cases, with other notable features being placental infarction, calcification, and chorioamnionitis. Placental findings were correlated with clinical causes of stillbirth. Findings in clinically unclassified stillbirths are also enumerated.

Conclusion: Our study underscores the importance of conducting autopsies and examining placental histopathology to determine the causes of stillbirth. Such insights are essential for planning personalized care, helping to improve outcomes in future pregnancies, particularly those following a loss.

## Introduction

Stillbirths and neonatal deaths are a global problem, with more than 6.4 million deaths occurring each year [1]. A baby born without any signs of life is called a stillborn. There is no universally accepted definition for stillbirth because of the differences in reporting policies in different countries and because of differences in terms of how the gestational age is assessed and interpreted [2]. The Royal College of Obstetricians and Gynecologists (RCOG) defines stillbirth as fetal death occurring during pregnancy at 24 weeks of gestation or later [3]. The definition recommended by the World Health Organization (WHO) for international comparison is ‘a baby born with no signs of life at or after 28 weeks' gestation’ [4].

The placenta is often called the ‘daily diary of records’ of the maternal-fetal interface, and its pathology is attributed as a cause or contributor to stillbirth in a significant proportion of cases [5,6]. The placental examination provides valuable information to determine the cause of stillbirth. Changes like fetal and maternal vascular malperfusion, thrombosis, and delayed villus maturation are indicative. However, at times, the changes are non-specific, like fibrin deposition. Besides, a single patient might have multiple disorders, and so it becomes difficult to interpret the placental changes. Moreover, some placental changes tend to recur in subsequent pregnancies and may cause recurrent pregnancy loss as well [7,8].

The present study aimed to decipher and compare the pathological changes in the placenta, umbilical cord, and autopsy findings in different causes of stillbirth. We also try to determine if placental pathology could identify probable causes in the case of clinically unclassified stillbirth.

## Materials and methods

This is a cross-sectional study conducted in the Clean Labor Room and Septic Labor Room of the Department of Obstetrics & Gynecology in collaboration with the Department of Histopathology, Cytology, and Gynae Pathology in a tertiary care hospital of Northern India, between April 2021 and July 2022. All women with stillbirths at >24 weeks of gestation who were willing for pathological examination of the placenta and autopsy of the baby were included in the study. The study was approved by the Institute Ethics Committee (INT/IEC/2021/SPL59 Ref no NK/7210/MD/458) and carried out in accordance with the Declaration of Helsinki and the ICMR guidelines.

**Figure 1:**
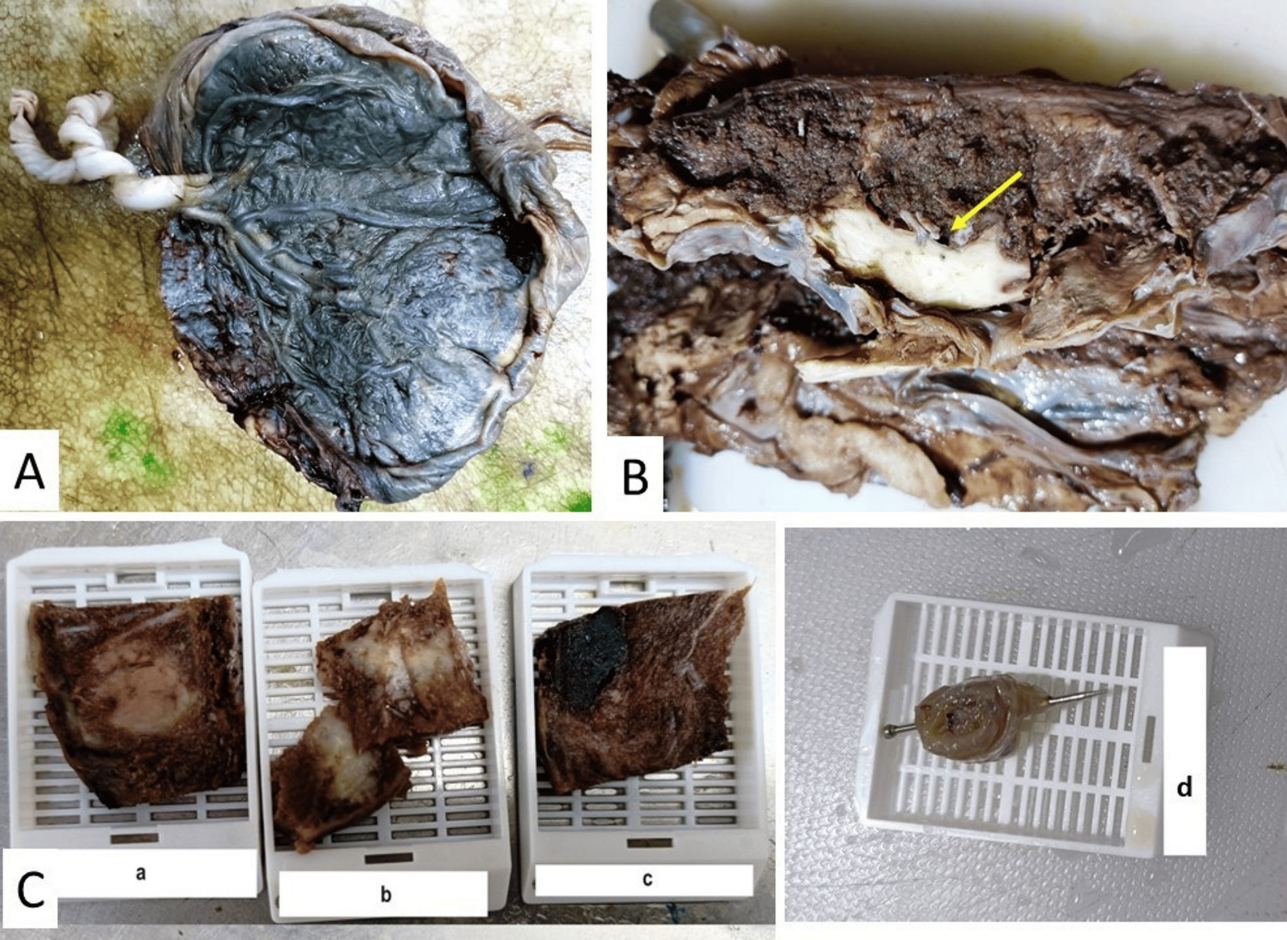
Gross photographs: A. Meconium-stained membranes B. Placental infarction (arrow) C. Sections submitted for embedding as per the Amsterdam Criteria: a and b, infarction; c, random section; d, Swiss roll of membranes

Intrauterine fetal demise (IUFD) was confirmed by an ultrasonogram by two clinicians/radiologists. A total of 50 subjects were enrolled. A detailed proforma was filled out to ascertain the cause and timing of stillbirth. A comprehensive history, including demographic details, history of present pregnancy, past obstetric history, and past and family history, was obtained. A detailed general physical, obstetric, and systemic examination was done. All antenatal investigations included blood tests (blood group, complete blood count [CBC], liver function test [LFT], renal function test [RFT], thyroid-stimulating hormone [TSH], glucose tolerance test [GTT], coagulation profile, culture, urine routine, microscopy, and culture), obstetric ultrasound reports, and aneuploidy screen. The probable cause of stillbirth was assigned based on clinical history and examination. Induction, monitoring, and labor analgesia were followed as per the departmental protocol. After delivery, the stillborn was weighed, and a detailed examination was done to identify any gross congenital malformations and the overall appearance of the baby with the help of a neonatologist. Thereafter, the pathologist did a detailed gross examination of the stillborn, placenta, membranes, and umbilical cord. The placenta was weighed and measured. Examination was done to look for the areas of infarct, retroplacental clot, accessory lobe, succenturiate lobe, bilobed, circumvallate, or circummarginate placenta, and to assess the site of cord insertion: central, eccentric, marginal, or velamentous. Sections were taken from each placenta as per the Amsterdam placental group workshop consensus statement [1] (Figure 1).

Sections included a roll of membranes, two transverse sections of the umbilical cord (one from the fetus and the other 5 cm from cord insertion), and three full-thickness sections of normal-looking placental parenchyma (including sections from the central two-thirds of the disc and adjacent to the insertion site). Grossly identifiable lesions were documented with measurement, appearance, number, and location, and sampled with at least one block. The umbilical cord was grossly examined for length, single umbilical artery, knots, strictures, cysts, and hematoma. The fetal weight, placental weight, and ratio of fetal weight to placental weight were noted. The stillborn was examined, and the length, weight, gender, nuchal cord, presence of gross malformations, and maceration were noted.

The final cause of stillbirth was assigned based on the autopsy and the histopathology report. The gross and histopathology findings were compared between different causes of stillbirths and the fetal weight, along with the placental weight. The ratio of fetal weight/placental weight was measured and compared between various causes of stillbirth. In cases where the cause of stillbirth was unclassified based on history and clinical examination, probable cause was determined clinically.

Data was analyzed using IBM Corp. Released 2016. IBM SPSS Statistics for Windows, Version 22. Armonk, NY: IBM Corp. and Microsoft Excel spreadsheets (Redmond, USA). The normality of all continuous variables, like age, etc., was checked using the Kolmogorov-Smirnov test. Data was expressed as mean ± SD for normally distributed continuous variables or median for non-normally distributed continuous variables and number (percentages for categorical variables). Student's t-test, Mann-Whitney U test, Fisher's exact test, chi-square test, and ANOVA were used, as applicable, to study the association of placental pathologies and the cause of stillbirth. A two-tailed p-value of ≤0.05 was taken as statistically significant with a 95% confidence interval.

## Results

In this cross-sectional study, conducted between April 2021 and July 2022, 50 women with a gestational age of 24 weeks or more and fulfilling the inclusion criteria were recruited.

The mean age of the enrolled women was 27.4±5.05 years, with 62% being primigravidae. Eighty percent of patients were seen in the BMI group 25-29.9 (80%). Most of the patients (97.2%) had a spontaneous conception. Only one patient had conceived by in vitro fertilization and had dichorionic diamniotic twins. Women suffering from stillbirth belonged more commonly to a rural population (Table 1).

**Table 1:** The demographic characteristics of the participants, obstetrical complications, and labor characteristics

Variables	Frequency (N=50)	Percent (%)
Geographical area		
Rural	31	62.00%
Urban	19	38.00%
Age(years)		
19-25	17	34.00%
26-30	17	34.00%
31-35	14	28.00%
>35	2	4.00%
Parity		
Primiparous	19	38.00%
2	19	38.00%
3	6	12.00%
4	6	12.00%
Gestation at presentation (weeks)		
24-28	9	18.00%
28-32	10	20.00%
32-36	16	32.00%
36-40	14	28.00%
>40	1	2.00%
BMI		
<18.5	0	0.00%
18.5-24.9	1	2.00%
25-29.9	40	80.00%
30-34.9	8	16.00%
>35	1	2.00%
Significant antenatal history		
Medications intake	7	14.00%
Leakage per vaginum	12	24.00%
Itching	9	18.00%
Fever	2	4.00%
Labor		
Spontaneous	31	63.20%
Induced	18	36.70%
Mode of Delivery		
Vaginal Birth	45	90.00%
Cesarean section	5	10%
Most likely attributable clinical cause of stillbirth		
Congenital malformations	12	24.00%
Unclassified	9	18.00%
Fetal growth restriction	8	16.00%
Hypertensive diseases in pregnancy	7	14.00%
Cholestasis of pregnancy	5	10.00%
Abruption	4	8.00%
Twin related	3	6.00%
Maternal disorder (acute fatty liver of pregnancy)	1	2.00%
Placental insufficiency with meconium aspiration	1	2.00%
Type of Stillborn		
Antepartum stillbirth	47	94.00%
Intrapartum stillbirth	3	6.00%
Macerated stillbirth	46	92.00%
Fresh stillbirth	4	8.00%

Two women each suffered from rheumatoid arthritis, Type II diabetes, and hypothyroidism. Four women had a history of previous stillbirths, one each attributed previously to diabetes, prematurity, hypertensive diseases in pregnancy, and placental insufficiency with meconium aspiration. Twelve women had significant malformations/soft markers of aneuploidy on fetal anomaly scanning, though none had aneuploidy screening done due to poor antenatal follow-up.

The most common probable clinical cause of stillbirths was fetal malformation (24%), while growth restriction contributed to 16%. Hypertensive diseases in pregnancy occur in 14% of pregnancies, cholestasis in 10%, abruption in 8%, and twin-related complications like twin-to-twin transfusion syndrome in 6%. The cause was unclassified in 18% (Table 1). Vaginal bleeding was the presenting complaint for three patients (6%), while leakage per vaginum was reported by 12 (24%) patients. Two patients had reported fever (4%), one prior to labor and the other had intrapartum fever. Labor was induced in 18 women, with hypertensive disorders of pregnancy being the most common indication for induction. The most commonly used agents for induction were prostaglandins. Out of 50, 45 (90%) had vaginal deliveries.

Fetal autopsy was sent for in all cases. However, most of the fetuses sent for autopsy (36/50) were autolyzed (Table 2).

**Table 2:** Pathological findings of placenta and fetus in stillbirths (n=50). Gross fetal malformations were limited due to extensive maceration in many babies DORV: double outlet right ventricle

	Frequency	Percentage
Gross fetal malformation		
None	42	84.00%
Anencephaly	2	4.00%
Hydrocephalus, hypospadias, shortened limbs	1	2.00%
Hydrops	3	6.00%
Low set ears	1	2.00%
Scaphoid abdomen, barrel shaped chest,	1	2.00%
Autopsy findings		
Autolysed, but no gross malformations	36	72.00%
Fetal Hydrops	2	4.00%
No congenital malformation	2	4.00%
Left renal agenesis	1	2.00%
Chorangiophagus Parasaticus twin	2	4.00%
Congenital diaphragmatic hernia	1	2.00%
Cephalhematoma	1	2.00%
B/L Primary Pulmonary Hypoplasia	1	2.00%
DORV	1	2.00%
Placental insufficiency with Meconium aspiration syndrome	2	4.00%
Amniotic fluid aspiration	1	2.00%
Total	50	100%
Macroscopic features of placenta		
Retroplacental Hematoma	4	8.00%
Infarction	6	12.00%
Microscopic features of placenta		
Villitis	1	2.00%
Accelerated villous maturation	0	0.00%
Delayed villous maturation	7	14.00%
Fibrin deposition	38	76.00%
Placental edema	0	0.00%
Thrombosis	1	2.00%
Calcification	13	26.00%
Infarction	14	28.00%
Distal villous hypoplasia	1	2.00%
Hyalinised villi	2	4.00%
Hydropic change	3	6.00%
Inter/intravillous hemorrhage	1	2.00%
Choriangosis	0	0.00%
Chorioamnionitis	12	24.00%

This could be explained by the fact that women with stillbirths are allowed to choose if they want termination of pregnancy or wish to wait for the spontaneous onset of labor. As in most cases, spontaneous onset of labor was preferred, which contributed to autolysis of the fetus before it reached autopsy examination.

**Figure 2:**
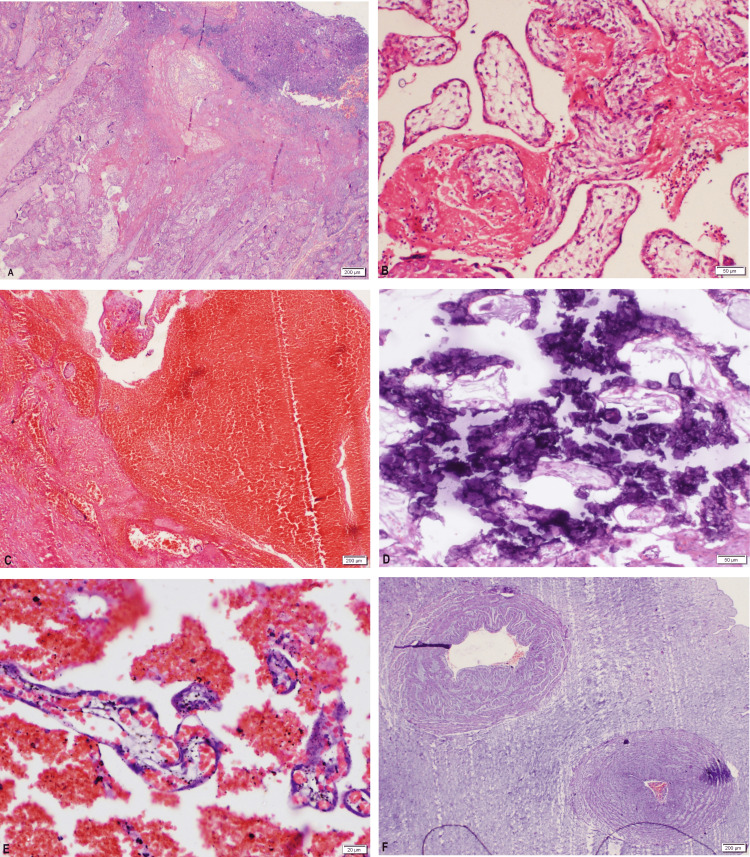
Microscopic images, placenta, and umbilical cord: A. Placental infarction B. Intervillous fibrin C. Retroplacental hemorrhage D. Calcification E. Intervillous hemorrhage F. Single umbilical artery

**Figure 3:**
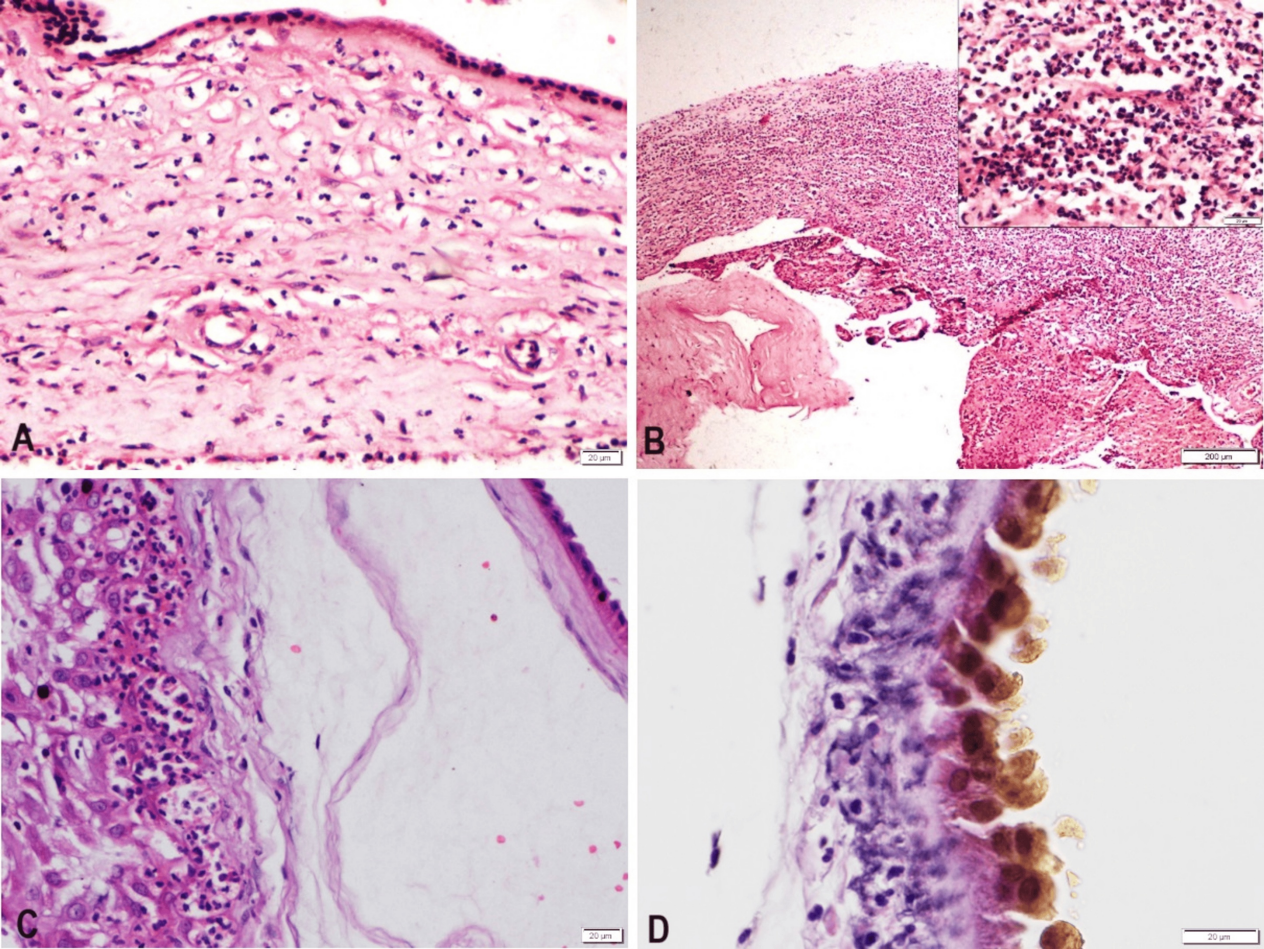
Microscopic images, membranes: A. Acute chorioamnionitis (Stage 2, Grade 1) B. Acute chorioamnionitis (Stage 3, Grade 2). The inset shows dense neutrophilic infiltrate at higher magnification. C. Acute chorionitis (Stage 1, Grade 1) D. Meconium staining of amniocytes

Placental examination was done as per the Amsterdam criteria (Figures 2, 3).

On gross examination, placental infarction was seen in six (12%) cases (four cases showing multiple infarcts, both peripheral and central, ranging from 1 cm to 3.5 cm in maximum dimension and involving between 15% and 40% of total placental volume, while two cases showed single peripheral infarcts measuring 2 cm and 2.5 cm in maximum dimension) and retroplacental hematoma, recent in four (8%) cases (with indentation of the placental parenchyma ranging from 3x2 cm to 6.5x4 cm). Marginal cord insertion was found in four (8%) patients in this study, and velamentous cord insertion was found in one (2%) patient. The most common pathological finding on placental microscopy was fibrin deposition, which was found in 38 (76%) of the cases. It was universally seen in all major causes of stillbirth, 91.6% of cases with fetal CMF, and 55% of cases with unclassified stillbirth (Table 3).

**Table 3:** Placental histopathology is seen in various prominent clinical causes of stillbirth

	CLINICAL CAUSE OF STILLBIRTH								
Placental Histopathology	Abruption	Congenital malformation	Fetal growth restriction	Cholestasis of pregnancy	Placental insufficiency with meconium stained liqor	Hypertensive diseases in pregnancy related	Twin related	Unclassified	Total
Villitis	0	0	0	0	0	0	0	1	1
Accelerated Villous maturation	0	0	0	0	0	0	0	0	0
Delayed villous maturation	0	2	4	0	0	0	0	1	7
Fibrin deposition	2	11	6	4	1	6	3	5	38
Villous stromal vascular karyorrhexis	0	0	0	0	0	0	0	0	0
Placental edema	0	0	0	0	0	0	0	0	0
Thrombosis	0	0	0	0	0	0	0	1	1
Calcification	2	2	2	1	0	4	0	2	13
Infarction	1	5	3	1	0	1	0	3	14
Distal villous hypoplasia	0	0	1	0	0	0	0	0	1
Hyalinised villi	0	0	0	1	0	1	0	0	2
Hydropic change	0	1	1	0	0	0	0	1	3
Inter/intra villous hemorrhage	0	0	0	1	0	0	0	0	1
Chorangiosis	0	0	0	0	0	0	0	0	0
Chorioamnionitis	1	2	1	1	0	1	2	4	12

Other common findings on placental histopathological examination were infarction and calcification, seen in 28% and 26% of all cases, respectively. Calcification was mostly found in pregnancies affected by hypertensive diseases in pregnancy (38%). Notably, chorioamnionitis was found in 12 cases (24%) of the total cases (maternal inflammatory response-stage 2 grade 1 [6 cases], stage 3 grade 2 [4 cases], and stage 1 grade 1 [2 cases]), out of which 6% had a history of leakage per vaginum at admission. Accelerated villous maturation, villous stromal karyorrhexis, placental edema, and chorangiosis were not found in any case.

The overall fetal weight/placental weight ratio of 50 cases was 5.8+/-4.2. In fetal growth restriction and hypertensive diseases in pregnancy-associated stillbirths, the mean FW/PW ratio was 5.3 +/- 3.4 and 6.6 +/- 1.92, respectively, while in diabetes with macrosomia, the fetal weight was disproportionately higher as compared to the placental weight, leading to a high FW/PW ratio of 19.6. In cases with congenital fetal malformations, it varies widely.

Nine (18%) of the total stillbirths were of unclassified causes. Histopathology revealed the presence of chorioamnionitis in 4 (maternal inflammatory response-stage 2 grade 1 [3 cases], stage 3 grade 2 [1 case]), hydropic change in one patient, calcification in two patients, infarction in three patients, fibrin deposition in five patients (Table 4).

**Table 4:** The major histopathological characteristics seen in cases of unclassified stillbirths FW: fetal weight, PW: placental weight

Gestation	FW (gram)	PW (gram)	Calcification	Infarction	Intramural fibrin deposition	Chorioamnionitis	Hydropic change
37	2700	520	+	-	+	-	-
24	445	170	-	-	+	-	-
38	3100	491	-	+	+	+	-
29	1200	647	-	+	-	+	-
34	1300	330	+	-	+	-	-
36	2400	240	-	+	-	-	+
30	505	270	-	-	-	+	-
31	1815	310	-	-	+	-	-
32	1060	200	-	-	-	+	-

## Discussion

This study was conducted in a tertiary care hospital in India, a part of Southeast Asia, a region that still contributes the highest number of stillbirths. Mothers are usually young, as in our study. The mean age of the mothers suffering stillbirth was 27.48 ± 5.05 years, which was comparable to the mean age in a previous study from India by Tiwari et al. [9]. However, most mothers were multiparous, in contrast to only 35% in the study [9]. Though various authors have shown that both low and high BMI were associated with an increased risk of stillbirth, in our study, all but one woman had a normal BMI, with only one patient with type 1 obesity. Most women presented late in the third trimester, though there were nine (18%) reported in the second trimester. A total of 47 (94%) out of 50 were antepartum stillbirths, mostly in women with unsupervised pregnancies. Previous stillbirth is associated with a higher risk of stillbirth, according to a study conducted by Gibbins et al. [10]. Four women (8%) of the women in our study had a history of stillbirth in a previous pregnancy.

The causes of stillbirths are many, ranging from fetal to maternal and placental. However, a large proportion of the stillbirths remain elusive with unknown causes. In the endeavor to reduce stillbirths, it is pertinent to explore the causes and take preventive measures to prevent their recurrence. The most common cause of stillbirth in our study was fetal malformation, accounting for 24% of cases, followed by fetal growth restriction and hypertensive disorders of pregnancy, which accounted for 16% and 14% of the cases, respectively. This contrasts with various other studies where the major cause was pre-eclampsia. This could be due to the higher referrals of malformed babies to our tertiary care institute that serves as a referral center to a large geographical area of northern India, and because parents of babies with malformations are more likely to consent to fetal autopsy, which can be an important inclusion criterion in our study. Though there has been a recent change in the law liberalizing the termination of pregnancy for fetal malformations detected later in pregnancy, during the period of the study, termination of pregnancy was limited to 20 weeks of gestation, thus inflating the numbers with respect to countries where fetuses with malformations are terminated and thus do not contribute to the stillbirth statistics. The most common fetal malformation in this series was nonimmune hydrops, which was seen in three patients (25%), in contrast to Tiwari et al., who reported neural tube defects, followed by nonimmune hydrops and cardiac defects, as the most common malformations in stillborns in their study (n=25, 16, and 11, respectively) [9]. More stillborn in our study were of male gender, like the findings demonstrated by Mondal et al., who found the risk to be 10% higher in males as compared to females [11]. The Y chromosome-linked genes are transcribed at the two-cell stage, and it was found that in animal models, male embryos have faster development and higher metabolic rates as compared to females, making the male fetus more susceptible to hormonal and oxidative stress [11].

Fetal autopsy is a useful investigation, but families often have many reservations about the same, including cultural, social, and personal reasons. Placental examination is a more acceptable option. Increasingly gaining focus, the placenta is often described as a black box, carrying known and unknown information about the causes of stillbirths. Research is ongoing, aiming to widen the knowledge on the role of placental pathology in deciphering causes of stillbirth [8]. The present study employed criteria as per the Amsterdam consensus placental workforce statement for histopathological examination of the placenta and for describing the lesions, which is being increasingly used to report placental pathologies [1,9].

The risk of stillbirth was higher in fetuses with a single umbilical artery, as it is usually associated with other congenital cardiac malformations [8]. In our study, a single umbilical artery (SUA) was found in three patients, two associated with fetal anencephaly and one with severe growth restriction. SUA is found in about one in 200 deliveries and is associated with chromosomal abnormalities, especially trisomy and multiple fetal defects [8]. SUA is also associated with fetal growth restriction [12]. Abnormal cord insertion is associated with a higher risk of stillbirth [13,14]. In our study, four (8%) patients had marginal cord insertion, out of which two patients (50%) had fetal growth restriction. Brouillet S et al. observed that fetal growth restriction was more common in those with peripheral cord insertion (20%) [12]. True cord knots are linked with an increased risk of stillbirth, and it is seen that the incidence of stillbirth is higher with multiple nuchal loops as compared to single nuchal cords [13]. The ratio of fetal weight to placental weight varies with different gestational ages and causes of stillbirth. The ratio is comparatively higher in diabetes due to a disproportionately large fetus compared to the placenta in cases of macrosomia. In our study, on gross examination, the placenta was ‘normal looking’ in about 80% of the cases.

Varied histopathological features in the placenta are associated with different causes of stillbirth. Some features may overlap between the various causes. Detailed histopathological examination of the placenta gives an insight regarding the cause of stillbirth as demonstrated by the current study [1,5,8,14-16]. In some instances, the placenta may give vital information to identify the cause, even if the autopsy fails to find the cause. Moreover, in certain cases, the causes of stillbirth are recurrent, and histopathological studies on the placenta may help in preventing preventable stillbirths [7].

The most common pathological finding on placental microscopy was fibrin deposition, which was found in 76% of the cases and seen in 91.6% of cases with fetal CMF. It was found in 55% cases with unclassified stillbirths. In one patient with meconium-stained liquor, this was the only pathological finding. According to a study by Lampi et al., the extent of fibrin seen in the placenta altered pregnancy outcomes and was associated with increased risk of prematurity, fetal growth restriction, and stillbirth based on the severity of fibrosis [15]. This contrasted with a study by Pinar et al., where the most common placental findings in stillbirths were inflammatory and thrombotic lesions [8]. The next common findings on placental histopathology in our study were infarction and calcification, seen in 28% and 26% of all cases, respectively. In a study by Purnima et al., infarction on microscopy was seen in 4.4% of preterm stillbirths and 13% of term stillbirths [9]. Calcification was most commonly found in women suffering from hypertensive disorders of pregnancy in our study (38%).

According to a population-based study conducted by Gordon et al. between 2007 and 2009, the incidence of histological chorioamnionitis in stillbirths was 22.6% in those with no clinical evidence of infection, causing both preterm and later-term stillbirths [17]. The proportion of stillborn babies with chorioamnionitis without a fetal inflammatory response who were classified as unexplained deaths was 53% (454/856), implying that histological chorioamnionitis may be associated with a significant number of unexplained stillbirths [17]. In our study, chorioamnionitis was found in 24% of the total cases (6% had a history of leakage, while in 18% of cases, there was no prior history of leakage), of which none had any clinical evidence of infection. Out of nine unclassified stillbirths in our study, four patients had histological chorioamnionitis. While one was a stillbirth at term, three were preterm. McClure et al. also demonstrated chorioamnionitis in 26.9% of stillbirths in their study from Southeast Asia [18]. Conclusively, placental examination may help identify the presence of subclinical chorioamnionitis in women with unclassified stillbirths [19].

Our study was not without drawbacks. It was an institution-based study at a single center and was limited by a lack of health controls. The study is limited to a small sample size. We only recruited patients who were willing to undergo both fetal autopsy and placental examination, contributing to recruitment bias, as a sizeable number of women/attendants refuse fetal autopsy due to religious or other reasons. Moreover, most women underwent expectant waiting for the spontaneous onset of labor, which led to autolysis of the fetal tissue and compromised the autopsy findings. The COVID pandemic and related restrictions also adversely impacted the feasibility of fetal autopsies and placental examinations, as well as the women suffering from stillbirths who consented to fetal and placental evaluation. However, being a tertiary care center, we had a dedicated gynae pathology department and expert gynae-pathologists available at our center, which may not be the case in the peripheral, rural, and far-flung areas.

## Conclusions

Our study highlights the critical value of autopsy and placental histopathology in uncovering the underlying causes of stillbirth. These investigations not only offer much-needed answers to grieving parents but also equip healthcare providers with essential insights. By understanding the cause, we can tailor care more effectively, ultimately guiding future pregnancies, especially rainbow pregnancies, toward safer outcomes and improved emotional and clinical support.
